# Telemedical Support in Patients with Chronic Heart Failure: Experience from Different Projects in Germany

**DOI:** 10.1155/2010/181806

**Published:** 2010-08-12

**Authors:** Axel Müller, Johannes Schweizer, Thomas M. Helms, Micheal Oeff, Claudia Sprenger, Christian Zugck

**Affiliations:** ^1^Clinic of Internal Medicine, Chemnitz Hospital, Bürgerstraße 2, 09113 Chemnitz, Germany; ^2^German Foundation for the Chronically III, Alexanderstraße 26, 90762 Fürth, Germany; ^3^Department of Internal Medicine I. Cardiology, Pulmonology, and Angiology, Municipal Hospital Brandenburg, Hochstraße 29, 14770 Brandenburg, Germany; ^4^Department of Internal Medicine III (Cardiology, Angiology, and Pneumology), University Medical Clinic, Im Neuenheimer Feld 410, 69120 Heidelberg, Germany

## Abstract

The great epidemiological significance and costs associated with chronic heart failure pose a challenge to health systems in Western industrial countries. In the past few years, controlled randomised studies have shown that patients with chronic heart failure benefit from telemedical monitoring; specifically, telemonitoring of various vital parameters combined with a review of the symptoms, drug compliance and patient education. In Germany, various telemedical monitoring projects for patients with chronic heart failure have been initiated in the past few years; seven of them are presented here. Currently 7220 patients are being monitored in the seven selected projects. Most patients (51.1%) are in NYHA stage II, 26.3% in NYHA stage III, 14.5% in NYHA stage I and only 6.6% in NYHA stage IV respectively. Most projects are primarily regional. Their structure of telemedical monitoring tends to be modular and uses stratification according to the NYHA stages. All projects include medical or health economics assessments. The future of telemedical monitoring projects for patients with chronic heart failure will depend on the outcome of these assessments. Only of there is statistical evidence for medical benefit to the individual patient as well as cost savings will these projects continue.

## 1. Introduction

In the Western industrial nations, chronic heart failure has great epidemiological significance due to the rising number of patients, particularly elderly ones [1]. This development occurs in addition to the demographic development characterised by low birth rates and an increasingly large proportion of old and very old people. According to the New York Heart Association (NYHA) stage, the prognosis for chronic heart failure is in some cases worse than for various malignant diseases [2].

Due to both, drug therapy (e.g., angiotensin-converting enzyme blockers, AT_1_-receptor blockers, aldosterone antagonists, or beta-receptor blockers) and medical devices (e.g., implantable cardioverter-defibrillators—ICD—and biventricular stimulation), the prognosis for chronic heart failure has improved in the past few years [3–5]. Therefore, these component of therapy have been incorporated into the guidelines of national and international specialist societies on the treatment of chronic heart failure [6, 7]. However, incorporating these recommendations, especially optimised drug treatment, into clinical practice has been unsatisfactory [5]. It has been shown that with targeted patient information and education, medication adherence and therefore medical outcomes of patients with chronic heart failure can be improved [8–10].

One of the primary goals of the treatment of patients with chronic heart failure is to avoid rehospitalisation. The number and duration of rehospitalisations due to decompensation of preexisting heart failure are not only of serious impact the patients quality of life, but also pose a large health-economic burden on the health system [11, 12]. The cost for in-patient treatment of patients with chronic heart failure in Germany amounted to 1.7 billion Euros in 2006. This corresponds to about 60% of the overall costs of therapy for heart failure [12].

**Figure 1:**
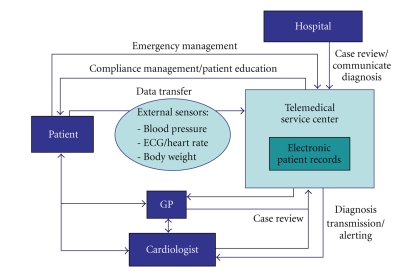
Overview of a complex telemedical support system for patients with chronic heart failure.

Thus, the primary goal must be to avoid or shorten renewed and prolonged rehospitalisations. Disease management programmes can reduce the mortality and rate of rehospitalisations and improve the quality of life of patients with chronic heart failure [13, 14]. In this context, questions arise regarding the options and efficiency offered by telemedical monitoring of patients with chronic heart failure. The definition of telemedical applications in this field is not uniform. The scenarios range from telephone support (nurse calls) and the monitoring of symptoms and compliance, to a complex kind of telemedical monitoring using automated transmission of data from medical devices [15–17]. The system used in the European Network Home Care Management System (TEN-HMS) study can now be considered the “classic scenario” for telemedical monitoring of patients with chronic heart failure [18]. This scenario is presented in a modified form in Figure 1.

In addition to the patients and the medical service providers (general practitioner, cardiologist in private practice, hospital), the most important partner in a complex telemonitoring system is the telemedical service centre. All data is collected here and integrated into the electronic patient records and constitutes the central core of case management. The patients transmit various vital parameters, either at intervals or continuously, to the telemedical service centre. The transmission of the vital parameters can be conducted via telephone or mobile phone. In Germany, one limiting factor seems to be the missing internet access, particularly in primary care practices.

The relevant monitoring parameters are heart rate, ECG (arrhythmias), blood pressure, and body weight. For instance, it was shown in a clinical study that atrial fibrillation worsens the prognosis for patients with chronic heart failure [19]. Body weight monitoring is an important clinical parameter in recognising cardiac decompensation. By regularly monitoring body weight, mortality was significantly reduced in the Weight Monitoring in Heart Failure (WHARF) trial [20]. An increase in body weight after release from hospital is an important predictor of rehospitalisation [21]. In addition, data from cardiac pacemakers, ICDs, or systems for cardiac resynchronisation (biventricular cardiac pacemakers or ICDs) are transmitted by remote control. The telemedical service centre monitors and selects the data. The data are then transmitted to the general practitioner, cardiologist, or hospital, as needed. The individual partners have access to the electronic patient records in accordance with the rules of data protection and with protected passwords. In addition, patients get contacted directly via telephone from the telemedical service centre. During these calls, questions can be asked about symptoms (edema, shortness of breath, etc.) and the medication taken. Furthermore, consultations and structured systematic training sessions on various problems related to heart failure are offered to patients. Thanks to a round-the-clock presence of doctors in the telemedical centre, it is possible to manage emergencies.

In the past few years, much international experience has been gathered in this regard on telemonitoring in patients with chronic heart failure, also in the context of randomised controlled studies. In the following, we report on selected projects in Germany.

## 2. Presentation of Individual Projects

The German health system has traditionally been structured according to sector, namely, the outpatient and inpatient sectors. Government policy has made various attempts in the last few years to overcome this division to some extent. One important mean to achieve this is contracts for integrated care [22].

In the past few years, a number of, mostly regional, model projects on telemonitoring patients with chronic heart failure have been conducted in Germany. These projects brought forth signs of cooperation between the hospitals and practising physicians, as the providers of medical care, and various health insurance organisations. In addition, providers of telemedical services and industrial partners were included in the cooperation. Table 1 provides an overview of selected telemedical projects for patients with chronic heart failure in Germany. 

Currently 7220 patients are being monitored in seven selected telemonitoring projects for patients with chronic heart failure. Most patients (51.1%) are in NYHA stage II, 26.3% in NYHA stage III, 14.5% in NYHA stage I, only 6.6% of patients were in NYHA stage IV, and in 1.6% of the Patients the NYHA stage was unknown.

The selected projects are presented in greater detail in the following sections.

### 2.1. “HeiTel”

The telemedical care of patients with heart failure in the context of the HeiTel project makes it possible, through monitoring, to institute and adjust therapy optimised for the individual patient. The patient transmits by telephone certain prescribed vital parameters (e.g., weight, blood pressure) via modem to the telemedical centre in an automated manner. If individually defined threshold values are not reached or exceeded, an alarm is immediately triggered in the monitoring centre so that therapeutic measures can be started immediately. Independent of any alarm responses, a patient in NYHA stages III-IV is contacted proactively at least once a week, while a stage II patient is contacted at least twice a month; they are asked questions according to a standardised form. The goal of these contacts is to promote medication adherence and to recognize telltale changes in a patient's health status as early as possible. Patients can reach the telemedical centre around the clock every day of the year to report cardiopulmonary symptoms and serious complaints. Training sessions on nutrition, movement and pharmacotherapy complete the programme and reinforce the patient's self-reliance in dealing with himself and his illness. 

After hospitalisation or after the conclusion of the individual titration phase during the initiation of drug therapy, which generally lasts about 6 months, there is a de-escalation of the device-based home monitoring, as part of a modular plan, which then becomes care provision by means of training sessions on nutrition, movement and monitoring of pharmacotherapy using a nurse call system. The purpose of this is to reinforce the patient's self-reliance in coping with himself and his illness and to perpetuate the success of treatment.

This modular plan offers the opportunity of establishing telemedical care as a cost-efficient module that is integrated into a chain of medical services. More than 200 general practitioners and cardiologists are currently actively participating in this plan.

The accompanying evaluation of the project documented a high-level of satisfaction among the participants. It moreover, also demonstrated the plan's efficiency in terms of health economics by means of a significant reduction both in the overall costs and in the number of days of hospitalisation for the HeiTel patients (*n* = 210) in comparison to a control group (*n* = 12 000) over a period of 2 years. This justifies the assumption that the intervention has a lasting effect. Although the plan was only designed for 12 months, these results led to an extension of the contract for integrated care.

### 2.2. The Telemedicine Centre Brandenburg (TMZB)

The Telemedicine Centre Brandenburg (Telemedizin Zentrum Brandenburg, tmzb) was established in 2004 by the Municipal Hospital Brandenburg in Brandenburg/Havel. Its aim is to improve the care for chronic cardiac patients in the federal state of Brandenburg. In cooperation with getemed, a medical technology company, and with funding by the European Union and by the Ministry of Economics of the state of Brandenburg a telemonitoring unit was developed which daily records and transmits all noninvasively parameters and information regarding clinical symptoms, drug compliance, and desired contact. 

The promising results of a self-financed pilot study led to a contract with the AOK Brandenburg (a state-licensed health insurance company) for integrated medical care for patients with chronic heart failure.

Cooperations with various hospitals were established, including the German Heart Centre in Berlin, and numerous hospitals in the state of Brandenburg, as well as consulting cardiologists and general practitioners. The telemedical monitoring allows surveillance of patients from far distance, which is advantageous in a large, sparsely populated state with a low density of physicians.

Inclusion criteria are chronic heart failure NYHA stages III-IV, an ejection fraction ≤40%, and a history of hospitalization for acute decompensated heart failure. At the moment, 300 patients of NYHA stages III and IV participate in the program.

The patients receive a telemonitoring device, developed by getemed, Teltow, a scale, ECG electrodes, and a blood pressure gauge. The patient daily measures weight, blood pressure, ECG, thorax impedance and breathing rate, and for certain indications also oxygen saturation. Patients also provide information regarding the severity of dyspnea, their subjective well-being, medication intake, and preference regarding desired personal contact. The data is encoded and transmitted by internet to the TMBZ, where it is analysed weekdays with regard to newly manifested arrhythmias, heart rate, body weight, and blood pressure using individually determined threshold values, breathing rate, and, if indicated, the oxygen saturation. The patient's subjective comments regarding dyspnea, peripheral edema, well-being, and drug compliance are evaluated to detect worsening symptoms. 

If deterioration has been detected, the patient's physician is informed by fax or, in urgent cases, via telephone by the tmzb. A summary of the reported results is included. This objective data enables better adjustment of the patient's medication according to individually changing health status.

The patient is contacted by the tmzb if: (i) suspicious changes of parameters are recorded, (ii) the patient requested contact, (iii) no data was transmitted, or (iv) the preceding intervention was not successful. 

The closer collaboration between the individual patients and their individual care givers (patient, general practitioner, cardiologist, hospital, telemedical centre) allows for an optimal care. The acceptance of telemonitoring among patients is very high, as indicated by the fact that the compliance with data transfer is 95%. Patients indicate that they feel secure. No serious technical problems have been encountered. Preliminary analyses of the data show a significant reduction both in number and days of hospitalisations for acutely decompensated heart failure [23].

### 2.3. “CorBene”

The “CorBene” project was developed as a contract for providing integrated care between practicing cardiologists, the Ford and Rhineland company health insurance programme, and the companies Vitaphone (Mannheim) and Medtronic (Düsseldorf). The goal is a transsectoral, stage-specific therapy of patients with chronic heart failure that is based on telemonitoring and is conducted according to guidelines. The participating doctors profit from a uniform documentation, improved exchange of information, and integrated quality management. This prevents, for example, redundant examinations. The telemedical concept of monitoring consists of four modules. This guarantees that the telemonitoring is appropriate to the NYHA stage and is individual. In module 1, the patient is given an ECG monitoring card to record his heart frequency and detect cardiac arrhythmias. Module 2 consists of weight monitoring. ECG and weight monitoring are linked together in modules 3 and 4. All reported data is integrated into an electronic patient record by the telemedical service centre Vitaphone, which is certified according to ISO and the Association for Engineering/Electronics/Information Technology (VDE) rules of application for telemonitoring [23, 24]. In addition, the staff of the telemedical service centre contacts the patient by telephone regularly, on a daily basis if needed. They enquire about symptoms that suggest imminent cardiac decompensation. There is also a reminder function for taking medication. Emergency management, including alerting the rescue services, is provided, made possible by the round-the-clock presence of physicians at the telemedical centre.

The “CorBene” project is being evaluated in terms of health economics. Initial data from 306 patients show that 81.2% of the patients included in the project are being treated according to the guidelines. 10.8% of these patients show clear improvement of clinical symptoms since they have been included in the project [25]. The final results of the health economics evaluation of the project are not yet available.

In the meantime, “CorBene”, which initially was a regional project, has been extended to cover all of North Rhine Westphalia (NRW) and Saarland. Currently more than 2900 patients with chronic heart failure are monitored by the project.

### 2.4. “Telemedicine for the Heart”

Integrated concepts of providing medical care that utilize telemedicine have been proven to be appropriate instruments for improving current deficits for patients with cardiac failure. For this reason the Techniker Krankenkasse health insurance company and the German Foundation for the Chronically Ill developed the integrated programme of medical care called “Telemedicine for the Heart”. Patients from throughout Germany with NYHA stages II-IV who are insured with the Techniker Krankenkasse have profited from this programme since January 1st, 2006.

The modularly structured programme for providing care, collecting data, interpreting data and training patients allows the participants to deal with their illness in a safer more self-empowered manner. It is also an important adjuvant to the treatment provided by their physicians. The goal of the patient-centred programme is to help the participating patients gain patient empowerment, to understand their illness better, to make the influencing factors between chronic heart failure, the prescribed medicine and their own life style more transparent. It also increases patient awareness of warning signs and symptoms, that indicate imminent decompensation. The analysis of life style with reference to the patient's own health situation encourages participants to reduce risk factors and expand health-promoting factors. By means of close coordination with the locally responsible physician, the programme aims to reduce the frequency and length of inpatient hospitalisations, at decreasing mortality, and at offering participants coordinated and high-quality medical care [26]. 

The programme is set to run for 27 months, which are divided into three phases. The first 6-month is the training phase. It familiarises the patients with the procedures of the programme and, by means of telephone-based training, provides specific knowledge about the complex topics associated with chronic heart failure. The second phase is the 3-month stabilisation phase. It promotes a consolidation of the behavioural patterns taught during the first phase and attempts to teach how to deal with the illness independently on a daily basis. The third phase consists of an 18-month refresher phase, aiming to anchor the behavioural patterns that promote better health in daily routines of participating patients, through constant repetition. This should allow, the results that have been achieved to be maintained after the participants have left the programme. The care and training given to patients is provided by a telemedical service centre (in this programme, Vitaphone). An electronic record is maintained for each patient at the telemedical service centre. For patients in NYHA stages III and IV, telemedical monitoring of body weight, blood pressure, and the pulse is also planned. During the programme, necessary interventions are made by the telemedical service centre team in coordination with the general practitioners and/or the cardiologists if a threshold is crossed.

In the context of a health economics evaluation conducted by the chair of health management at the University of Erlangen-Nuremberg, the data of 281 participants in the programme “Telemedicine for the Heart” (treatment group) were compared to the data of a matched control group that was three times larger in size. Examination of the number of hospitalisations per patient and year, showed that for the participants in the programme there were 21.5% fewer hospitalisations (*P* = .03, *t*-test). This means that it was possible to avoid approximately every fifth hospitalisation as the result of this intervention (i) an improved level of knowledge among the participating patients, (ii) an early warning function of the telemedical monitoring, and (iii) close coordination between the telemedical centre and the responsible physicians. 

The evaluation also provides a clearly positive result with regard to mortality. In the group of programme participants, there was a 51.7% (*P* = .01, chi-squared test) reduction in mortality within the first year, that is, that with the most intensive training and support phases. Even if the analysis is extended to the maximum period of time (including the last phase of the programme with a low intensity of care provision), the mortality was reduced by 35.1% (*P* = .04, chi-squared test), a value more than one third lower than the mortality in the comparison group.

In the context of the evaluation, it could be shown that the programme participants had a uniformly higher supply rate of heart-specific medication. The training sessions focused on heart failure and the communication optimised within the integrated care context met the programme's goal of promoting the prescription of diagnosis and guideline specific drugs. 

The medical effects reported above were achieved in a cost-efficient manner, as is shown by the overall costs. There are substantial differences in favour of the treatment group, both after 1 year of programme membership (savings of 1592.16*€*, 18.1% of the total costs) as well as over the maximum period of time observed normalised per year (savings of 2633.40*€*, 25.0% of the total costs). These data make it clear that the complete 27-month programme fully paid for itself in its first year of work, while the effects of the care provision—especially the effect of the training sessions—were maintained over a much longer period of time.

In summary, the evaluation showed that the optimised interplay of therapeutic supervision by the local physician with the supportive care and training sessions provided by “Telemedicine for the Heart” offers an important contribution to stabilising patients' health status. This programme is able to train the participants in a cost-efficient manner. They learn to deal with their illness and to recognise imminent decompensation early, especially if using specific telemedical technology and the close coordination with their physicians. This allows for the adjustment of therapy even on an outpatient basis, lowering mortality, and avoiding repeated and cost-intensive hospitalisations.

### 2.5. “Partnership for the Heart”

The research and development project “Partnership for the Heart” is jointly sponsored by the Charité, University of Medicine Berlin, the Robert Bosch Hospital in Stuttgart, and three industrial partners. This project receives support from the government agency for economics and technology. 

The aim of the project is to establish a sensor platform for patients, an electronic record for patients, as well as a telemedical centre [27]. The sensor platform is a wireless network (local area network) in the patient's home. Various sensors (for measuring blood pressure, a three-channel ECG, weighing scales, and an activity sensor) are integrated into this network. The sensors are connected wirelessly via Bluetooth to a PDA that is part of a mobile phone network. The measured data is ultimately transferred to the telemedical center via the PDA. At the telemedical center, the data is integrated into the patient's electronic record. The telemedical centres are directly attached to the hospitals (Charité Berlin, and Robert Bosch Hospital in Stuttgart) and are staffed by physicians and caregivers. The staff also has the contact data of the patients' general practitioners and specialists and the phone numbers of the regional emergency services [27]. 

The Partnership for the Heart study has examined a total of 710 patients since January 2008. It is a multicentric randomised prospective study of patients with chronic heart failure that uses an open and controlled design (telemedical participants and a control group) and follows patients for a period of at least 12 months. In the telemedical group, there is daily monitoring of blood pressure, ECG, oxygen saturation, and body weight. Furthermore the patients' physical activity is evaluated by an activity sensor and the patient himself evaluates his own health status. The study is financed and supported logistically by the Barmer and Bosch health insurance companies. The primary end point of the study is the period of survival. Secondary end points of the study are overall mortality, cardiovascular mortality, frequency of any kind of nonelective hospitalisation, frequency of hospitalisations for cardiovascular reasons, the plasma level of NT-proBNP over time and the quality of life over time, An evaluation from the perspective of health economics is planned [27, 28].

### 2.6. “HeartAs/HerzAs”

The project “HeartAs” was established in combination with an integrated-care contract. Until now, 516 patients have been included in the project, 96 of which have already concluded the program. The majority of patients are in NYHA stage II and III. In this project, body weight, ECG (12-channel or 1-channel), pulse, and blood pressure are transmitted. The project consists of a modular character. In addition to compliance management and emergency management modules, regular telephoneadvice calls are given. A medical, academic, and economic (cost benefit analysis) evaluation will follow.

### 2.7. “ProHeart/Herzensgut” 

Some initial experience regarding the telemedical monitoring of patients with chronic heart failure has been gathered in a project organised by the health insurance company “Kaufmännische Krankenkasse” and the Almeda company in Munich. The idea behind this project was similar to that of the ProHeart project. Participants were contacted regularly by telephone by specially trained medical personnel. On the one hand, the use of specially developed software meant that the telephone conversations were very standardized, while on the other, there was always an opportunity to discuss the patient's situation in detail in an individual and problem-oriented manner. The telephone contacts were supplemented by written training material [29]. There was, in addition, telemetric monitoring of the patient's weight. Compared to a control group with 183 patients, it was possible to lower the number of days in hospital by 48% in the group of participants (214 patients). An overall reduction in cost of 39.5% was achieved. The evaluation was conducted in a stratified manner over a 1-year period, while the observation period was in fact at least 6 months and at most 18 [30].

During the observation period, 14.7% of the participating patients died, compared to 27.1% in the control group. This difference was statistically significant [30].

The project “ProHeart” is currently conducted by Almeda company and the Allianz health insurance. Up to now, 1545 patients in NYHA stages I–IV have been included in the project

## 3. Discussion

The German projects on telemedical monitoring of patients with chronic heart failure that are described individually above exhibit different approaches and plans for improving the situation of these patients. Nonetheless, they share some fundamental strategies. The goals are, besides monitoring the patients' status (weight, blood pressure, ECG), to remind patients of their medication, to monitor symptoms indicative of imminent cardiac decompensation, and to train patients on topics relevant to heart failure. A significant concern of all the programmes is increasing the ability of the patients themselves to manage their own illness. Most programmes are planned for a limited time period. The timely limitations of this program have to be discussed critically. Most of the projects described were established and conducted within the confines of integrated-care-contracts over a predefined frame of time. Undisputed is the fact that chronic heart failure can improve only with consistent surveillance, for which reason patients need further long-term monitoring. This poses the question of which patients (NYHA stage) derive the most benefit from telemonitoring and which intensity (daily, weekly…monitoring) should be adjusted to individual patient needs. As a foundation, consistent definition and measures of goals needs to be defined (mortality, rehospitalisation, quality-of-life). The discussed projects demonstrate the heterogeneous of patients included according to differences in NYHA stages. 

In a meta-analysis of 14 randomised controlled studies on telemonitoring of patients with heart failure, Clark et al. demonstrated there was a 20% reduction in overall mortality [17]. The effect was greater in the telemonitoring programmes than in the structured telephone. It is essential to distinguish between the structured telephone support and the telemonitoring programmes with transfer of vital parameters (body weight, heart frequency, blood pressure, ECG) [17].The effects were greater in the telemonitoring programmes than in the structured telephone support programmes, although the difference was not statistically significant. Furthermore, the rate of rehospitalisation for cardiac decompensation was reduced by 21%. Here, too, there was no difference between the two groups [17]. 

Roth [31] and associates could demonstrate a reduction of total hospital days of 66% with the usage of telemonitoring in patients with chronic heart failure, in the SHL Telemedicine Projects. Most patients reported a significant improvement of their quality-of-life [31].

The results clearly show that telemonitoring and telephone support programmes are beneficial with regard to mortality and the rehospitalisation rates for patients with chronic heart failure [15–17]. The central question continues to be the nature of the intervention that the patient requires. The results of the TEN-HMS study show that the results for mortality and rehospitalisation were similar in both groups. The programmes in the device-based telemedicine branch, however, were more cost efficient than the other programmes even after the system-related additional costs for the devices were included, since it was possible to shorten the length of in-patient treatment [18]. This makes a differentiated use of telemedicine necessary for individual patients. The range of items to be monitored should be selected according to NYHA stage and be modular. For most patients in NYHA stage II, a telephone support programme with monitoring of symptoms, compliance management, training, and facilitating self-observation appears to be sufficient in most cases. Patients in NYHA stages III and IV would benefit more from telemonitoring with the additional daily transfer of vital parameters. The telemedical monitoring software should also have an individual character. In practice, this means that only those parameters relevant for the individual patient (e.g., body weight, blood pressure) are monitored. At the patient's end, these parameters are transmitted, for instance, using Bluetooth via the individual devices (such as weighing scales, blood pressure gauge) to a central patient monitor. The patient monitor transmits the data on to the telemedical service centre, generally via landline. Currently, it depends on the telemedical provider involved which of the different hardware components are available. The systems are not compatible with each other. This means that a modular structure of heart failure programmes that accords with the NYHA stages is an indicator of quality in such programmes. In Table 2, the quality requirements on telemedical monitoring programmes for patients with chronic heart failure are summarized. In two current randomised studies on telemonitoring [Home or Hospital in Heart Failure (HHH) Study and Home Heart Failure (HF) Study] could show evidence of a high degree of compliance in data transmission even in older patients [32, 33]. However, the use of telemedical monitoring in the HHH Study was unable to significantly reduce the duration of in-patient treatment for cardiac failure, cardiac death and hospitalisation due to heart failure in comparison to usual treatment. Similar results were reported in the Home HF Study [33].

The reasons discussed for this include the high level of standard treatment for heart failure and intermittent data transmission (once a week) [32]. The telemedical projects conducted in Germany monitor a variety of vital parameters (e.g., weight monitoring only or complex monitoring of weight, ECG, blood pressure, pulsoximetry) and define various monitoring intervals (daily or weekly). In order to be able to compare the results, standardisation seems necessary (choice of vital parameters to monitor, transmission intervals, automatic data transmission; Table 2).

The electronic patient record is the central component of telemedical patient monitoring. Recognising deviations from established alarm thresholds allows both patients and the treating physician (general practitioners, cardiologists) to be alerted promptly. This permits close coordination between the partners and early intervention in the patient. In addition, personal telephone contact to the patient is established through the telemedical service centre. By means of this, enquiries can be made about symptoms and complaints, it can be checked whether medication is being taken, and patient education can be coordinated. The quality of the telemedical care systems depends significantly on the work of the employees in the telemedical service centre. The aim here must be to standardise procedures and certification of the telemedical service centres in the context of quality assurance (Table 2). To this end, uniform standardized operating procedures (SOPs) must be defined and put into practice. In Germany, telemedical support is provided by specialized telemedical service centres. These may be associated with individual Hospitals or be commercial providers (i.e., SHL Telemedicine, Vitaphone, or Almeda). In these telemedical service centers, specially trained personnel contacts patients and physicians, monitors the vital signs, and maintains the electronic patient record. The association for Engineering/Electronics/Information Technology e.V. (VDE) has established guidelines for telemonitoring and certifies telemedical service centers [24, 34]. This is the first step toward standardization and comparability of different programs. 

Another essential concern in telemedical monitoring is intercommunication between general practitioners, cardiologists in private practice, hospitals and rehabilitation facilities. This aspect is of great importance in Germany since here there is traditionally a great division between outpatient and inpatient sectors. Most telemedical monitoring programmes were conducted for this reason in the context of contracts for integrated care. In doing this, in addition to physicians, medical insurance companies and telemedical providers would be directly included in the projects.

The greatest challenge at the moment is the reimbursement of telemedical services. This applies both to the service providers (physicians in private practice, hospitals) and the telemedical providers. At the moment in Germany, telemedicine for monitoring patients with chronic heart failure is not a component of standard treatment. Thus it can also be explained why most projects are regional in character. In the next few years, it will be necessary to more strongly develop the evidence of the benefits of telemedical monitoring in patients with heart failure. For this, in addition to medical and economic evaluations (mortality, rehospitalisation rates, therapy according to guidelines, quality of life), health-economic evaluations are also necessary. The programmes presented here have this health-economic evaluation integrated into them as a fixed component of the programmes. Proof of the cost efficiency of the programmes will be a crucial point for the future of telemedical monitoring programmes. Currently, none of the described projects have been completed, so that final results are not yet available.

## 4. Summary and Perspective

In Germany, various projects on telemedical monitoring of patients with chronic heart failure could have been set up in the past few years. Most of these projects were initiated in the context of contracts of integrated care and are mostly regional in nature. The telemedical monitoring programmes differ in terms of their choice of monitored vital parameters, sequence and frequency of data transmission, and duration. This makes a comparison only possible to a limited extent. In addition, most of the programmes are not yet completed, so that no final assessment of the medical-economic or health-economic evaluation is yet possible. The further acceptance of patients and sponsors of projects on telemedical monitoring of patients with chronic heart failure will depend in the future on the evidence for improvement of the medical variables (mortality, hospitalisation rate, duration of hospital treatment) as well as evidence of cost efficiency. Standardization is required of the programmes for telemedical monitoring of patients with chronic heart failure according to defined quality criteria. 

For the context of further studies some basic questions, such as which patients (NYHA stage) derive the most benefit at which degree of intensity of telemonitoring, need to be considered, in order to achieve lasting improvements for these patients. In addition, attention need to be given to the affects of the physical separation of telemonitoring on the doctor-patient relationship in order to increase the physician's and patient's acceptance of telemonitoring.

## Figures and Tables

**Table 1:** Selected telemedical support projects for patients with chronic heart failure in Germany.

Project	HeiTel-Telemedicine	CorBene	Telemedicine for the Heart	Telemed Brandenburg	Partnership for the Heart	HerzAs	Pro Heart/Herzensgut
Partner	200 GP and specialists	Cardiologists, GP hospitals, Rehabilitation facilities, Medtronic GmbH	German Foundation for the Chronically III	Cardiologists, GP, local and regional hospitals, Deutsches Herz-zentrum Berlin	Bosch Telemedicine GmbH, Intercomponent Ware AG, getemed AG, T-Mobile GmbH	BNK Westfalen-Lippe	BNK Service GmbH, KKH Allianz

Associate partners	AOK Baden-Württemberg	Study groups of the Betriebs-Krankenkassen NRW/Saarland	Techniker Kranke kasse	AOK Branden-burg	Barmer Ersatz-kasse	AOK Westfalen-Lippe, KVWL-Consult	

Telemedical provider	SHL Telemedicine Düsseldorf	Vitaphone GmbH Mannheim	Vitaphone GmbH Mannheim	TMZ Brandenburg, getemed Teltow	Telemedical center Charite Berlin, Telemedical center Robert-Bosch-Hospital Stuttgart	Institute for applied telemedicine Bad Oeynhausen	Almeda AG München

Start of the project	2006.09.01	2005.12.01	2006.01.01	2004	2005.01.01	2008.01.01	2004.01.01

End of the project	2010.12.31	unlimited	unlimited	unlimited	2010.06.30	unlimited	until 12/2009

Number of patients	217	2928	1100	300	710	420*	1545

NYHA stage I	48	530	0	0	0	11	456

NYHA stage II	83	2062	627	0	312	116	488

NYHA stage III	85	302	429	195	398	174	317

NYHA stage IV	1	34	44	105	0	6	284

Duration	12 months	unlimited	6–27 months	at least 1 year	25 months	at least 1 years	2 years
Transmitted parameters	Body weight, ECG/heart rate, blood pressure	Body weight, ECG/heart rate, symptoms	Body weight, heart rate, blood pressure	Body weight, ECG/heart rate, blood pressure, thorax impedance and breathing rate, oxygen saturation, symptoms	Body weight, ECG/heart rate, blood pressure physical activity, Self perceptance	Body weight, ECG/heart rate, blood pressure	Body weight, heart rate, blood pressure

Modular system for telemedical monitoring	yes	yes	yes	limited	yes	yes	no

Compliance management	yes	yes	yes	yes	yes	yes	yes

Patient education program	yes	yes	yes	yes	yes	yes	yes

Emergency management	yes	yes (24/7)	limited	yes	yes (24/7)	yes	limited

Medical scientific assessment	yes	yes	yes	yes	yes	yes	no

Health-economic assessment	yes	yes	yes	yes	yes	yes	yes

Further informations	http://www.klinikum.uni-heidelberg.de/	http://www.vitaphone.de/	http://www.dsck.de/	http://www.tmzb.com/	http://www.partnership-for-the-heart.de/	http://www.hdz-nrw.de/	http://www.almeda.com/

(*in 113 patients the NYHA stage was not determined).

**Table 2:** Quality requirements for telemedical support programmes for patients with chronic heart failure.

(i) Monitoring according to the NYHA stage
(ii) Ensuring treatment according to guidelines
(iii) Creating a modular system for individual telemedical monitoring
(iv) Standardizing telemedical monitoring (selection of vital parameters, transmission intervals, automatic data transfer)
(v) Integrating the recorded data in an electronic patient record
(vi) Networking between partners (general practitioner, cardiologist in private practice, hospital, rehabilitation facility)
(vii) Certification of the telemedical service centre according to defined standards
(viii) Establishing standardised operating procedures (SOPs) in telemedical service centres
(ix) Establishing an emergency management plan
(x) Contacting the patient directly by phone for reviewing symptoms, medication adherence, consultation
(xi) Enabling the patient to be self-reliant
(xii) Accompanying patient education with structured training programmes
(xiii) Accompanying medical and health-economic assessment
